# Comparison of Moderate Hypofractionated Volumetric-Modulated Arc Therapy Plans With and Without Flattening Filter for Localized Prostate Cancer

**DOI:** 10.7759/cureus.18034

**Published:** 2021-09-16

**Authors:** Yosuke Takakusagi, Keisuke Usui, Nobutaka Mizoguchi, Jun Nagatsuka, Takeshi Hikage, Yasuhiko Kodama, Takatomo Ezura, Terufumi Kusunoki, Yukio Oizumi

**Affiliations:** 1 Department of Radiation Oncology, Kanagawa Cancer Center, Yokohama, JPN; 2 Department of Radiation Oncology, Juntendo University, Tokyo, JPN; 3 Department of Radiology, Yokosuka General Hospital Uwamachi, Yokosuka, JPN; 4 Section of Medical Physics and Engineering, Kanagawa Cancer Center, Yokohama, JPN; 5 Department of Radiation Oncology, Yokosuka General Hospital Uwamachi, Yokosuka, JPN

**Keywords:** comparison, moderate hypofractionation, prostate cancer, vmat, flattening filter-free

## Abstract

Background/Aim

The aim of this study was to compare volumetric-modulated arc therapy (VMAT) radiation plans between conventional VMAT with flattening filter (cFF-VMAT) and flattening filter-free VMAT (FFF-VMAT) for localized prostate cancer.

Materials and methods

Ten patients with localized prostate cancer who underwent cFF-VMAT at Yokosuka General Hospital Uwamachi, Yokosuka, Japan, from July 2020 to October 2020 were enrolled. Dose-volume histogram (DVH) parameters of the target volume, normal organs, monitor units (MU), and beam-on time (BOT) were compared between cFF-VMAT and FFF-VMAT plans.

Results

No significant difference was observed for DVH parameters for the target volume. No significant difference was observed in all parameters for the bladder and rectum between the cFF-VMAT and FFF-VMAT groups. The mean values of MU were 686 ± 52 and 784 ± 80 in cFF-VMAT and FFF-VMAT, respectively (p < 0.001). The mean BOT was 97.0 ± 6.6 s and 72.9 ± 1.4 s for cFF-VMAT and FFF-VMAT, respectively (p < 0.001).

Conclusion

DVH parameters of the target volume and normal organs were not significantly different between the cFF-VMAT and FFF-VMAT plans. In FFF-VMAT, MU was significantly higher, and the BOT was significantly shorter than those in cFF-VMAT.

## Introduction

Prostate cancer is the second most common cancer worldwide and the fifth based on mortality rate [1]. Radiation therapy (RT) is one of the radical treatment modalities for localized or locally advanced prostate cancer. Favorable treatment outcomes have been reported by escalating the radiation dose [2-5]. Development of radiotherapy techniques, such as intensity-modulated radiotherapy (IMRT) and volumetric-modulated arc therapy (VMAT), can deliver dose escalation to the target volume without increasing the toxicity to surrounding normal organs [2]. Therefore, IMRT is widely used worldwide [6,7].

The α/β ratio of prostate cancer has been suggested to be very small [8-12]. Therefore, hypofractionated RT with a higher dose fraction was expected to demonstrate a favorable therapeutic effect. In fact, several randomized controlled trials on the use of hypofractionated RT for prostate cancer have been conducted and found to be comparable to the conventional fractionated RT [13-15]. Conversely, irradiation time generally becomes longer in hypofractionated RT. Intrafractional motion has been found to be increased as the irradiation time becomes longer [16].

Flattening filter-free (FFF) beams can provide higher dose rates and shorter irradiation times than flattening filter beams [17]. In several studies on FFF-VMAT for prostate cancers, FFF-VMAT has been reported to have similar dose distribution and shorter irradiation time compared to the conventional VMAT with flattening filter (cFF-VMAT) [18-21]. These studies demonstrated that ultrahypofractinated RT with a large dose fraction or conventional fractionation was applied for the treatment of prostate cancer. Although dose distribution and technical parameters of treatment may differ depending on dose fractionation, only rare studies have compared cFF-VMAT and FFF-VMAT in moderate hypofractionated RT for prostate cancer. Therefore, this study aimed to compare the moderate hypofractionated treatment plans of cFF-VMAT and FFF-VMAT for localized prostate cancer and quantitatively evaluate the dose distribution and technical parameters in irradiation.

## Materials and methods

Patients

This study enrolled 10 consecutive patients who underwent cFF-VMAT for localized or locally advanced prostate cancer at Yokosuka General Hospital Uwamachi, Yokosuka, Japan, from July 2020 to November 2020. The study was approved by the Institutional Review Board of Yokosuka General Hospital Uwamachi (approval number: 2020026).

Treatment planning

Patients were placed in the supine position and immobilized with heel support (Engineering System, Nagano, Japan). The patients urinated and drank water 60 min before undergoing computed tomography (CT) scan. CT was performed with 2-mm slices. Gross tumor volume was not delineated. Clinical target volume (CTV) included the entire prostate and proximal seminal vesicle. The planning target volume (PTV) was extended 5 mm in all directions from the CTV, except at 3-mm posterior margin. The rectum and bladder were delineated as organs at risk (OAR). The rectum was delineated from the anal canal to the rectosigmoid flexure. The same structure was used for cFF-VMAT and FFF-VMAT.

All treatment plans were analyzed retrospectively. The total dose was set at 70 Gy in 28 fractions and was prescribed as the mean dose (Dmean) of PTV [22]. Dose constraints for normal organs were as follows: percentage of the rectum volume receiving at least 37 Gy (V37) < 50%, V60 < 18% for rectum, and V60 < 40% for bladder. Among these dose constraints, the equivalent dose in 2 Gy per fraction (α/β = 3) was 32.0 Gy and 61.7 Gy, respectively. Treatment plans were generated using Monaco version 5.11 (Elekta AB, Stockholm, Sweden). Treatment plans were created and reviewed by two or more radiation oncologists, radiation technologists, and medical physicists. To avoid bias in the creation and assessment of treatment plans, all treatment plans were created using the same plan template. A 10-MV (mega-electron-volt) x-ray was used. The maximum dose rate was 600 monitor units (MU)/min for cFF-VMAT and 2200 MU/min for FFF-VMAT. VMAT plans used one full arc. The collimator angle, increment angle, and calculation grid were set at 10°, 30°, and 2 mm, respectively. The calculation algorithm used was the x-ray voxel Monte Carlo method. Uncertainty per calculation was set at 1.0%. All treatment plans were transferred to the MIM maestro software version 7.0 (MIM Software Inc., Cleveland, USA), and dose-volume histogram (DVH) parameters were estimated. The following DVH parameters were assessed: the dose covering 98% of the target volume (D98), D95, D50, D2, homogeneity index (HI), conformity index (CI), and mean absolute dose deviation (MADD) for PTV. HI was calculated using (D2-D98)/D50 [23]. CI was calculated using V95/VPTV [23]. MADD was calculated as:

$MADD=\int_{0}^{V0}\frac{\left | D-A \right |}{V0}dV$ [24].

The notation was applied as follows: D is the dose, and V is the volume ordinate of the set of points representing the cumulative DVH curve of the structure, V0 is the volume of the structure, and A is the reference dose for the structure.

V95 represented the volume irradiated with 95% of the prescribed dose. VPTV represented the PTV volume. V10, V20, V30, V40, V50, V60, and Dmean were evaluated for the bladder and rectum.

MU and beam-on time (BOT) were investigated as technical parameters. The irradiation was performed using Axesse (Elekta AB, Stockholm, Sweden). BOT was measured by pressing the start button until the beam went off. BOT measurements were performed three times, and the average time was used.

Statistical analysis

DVH parameters for each treatment plan were compared using the Wilcoxon signed-rank test. The correlation between the PTV volume and technical parameters was evaluated using the Pearson correlation coefficient. A weak correlation was defined as r < 0.4, moderate correlation as 0.4 ≤ r ≤ 0.7, and strong correlation as r > 0.7. A p-value of <0.05 was considered statistically significant. Statistical analysis was performed using the STATA software version 13.1 (StataCorp, College Station, USA).

## Results

Planning target volume

**Figure 1:**
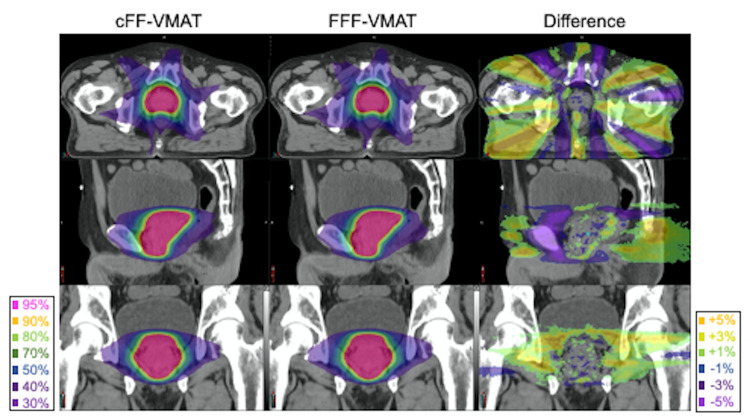
Comparison of typical dose distribution The image shows the dose distributions of conventional volumetric-modulated arc therapy with flattening filter (cFF-VMAT) and VMAT with flattening filter-free beams (FFF-VMAT). The dose difference distribution map between cFF-VMAT and FFF-VMAT was also demonstrated.

**Figure 2:**
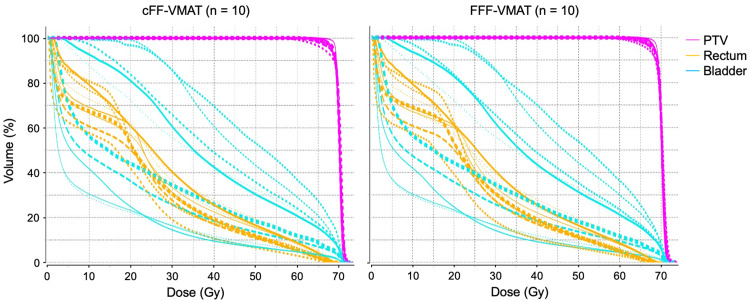
Dose-volume histogram of all cFF-VMAT and FFF-VMAT plans VMAT, Volumetric-modulated arc therapy; cFF-VMAT, conventional volumetric-modulated arc therapy with flattening filter; FFF-VMAT, VMAT with flattening filter-free beams; PTV, planning target volume.

Figure 1 shows a typical dose distribution and DVH in the cFF-VMAT and FFF-VMAT plans. Figure 2 shows all DVH in the cFF-VMAT and FFF-VMAT plans. DVH parameters for PTV are summarized in Table 1. No significant difference was observed for DVH parameters in the cFF-VMAT and FFF-VMAT plans. The mean values of HI were 0.08 ± 0.02 and 0.08 ± 0.02 for cFF-VMAT and FFF-VMAT, respectively (p = 0.170). The mean values of CI were 1.27 ± 0.09 and 1.25 ± 0.08 for cFF-VMAT and FFF-VMAT, respectively (p = 0.981).

**Table 1:** Dosimetric comparison for PTV between cFF-VMAT and FFF-VMAT VMAT, Volumetric-modulated arc therapy; cFF, conventional flattening filter; FFF, flattening filter-free; PTV, planning target volume; DVH, dose-volume histogram; MADD, mean absolute dose deviation; SD, standard deviation.

DVH parameters	cFF-VMAT (mean ± SD)	FFF-VMAT (mean ± SD)	p-value
PTV			
D98 (Gy)	66.2 ± 1.6	66.2 ± 1.4	0.647
D95 (Gy)	68.1 ± 0.7	68.0 ± 0.6	0.541
D50 (Gy)	70.2 ± 0.1	70.2 ± 0.1	0.799
D2 (Gy)	71.5 ± 0.2	71.6 ± 0.3	0.146
Homogeneity index	0.08 ± 0.02	0.08 ± 0.02	0.445
Conformity index	1.27 ± 0.09	1.25 ± 0.08	0.058
MADD (Gy)	0.38 ± 0.09	0.40 ± 0.09	0.525

Organs at risk

DVH parameters for OARs are summarized in Table 2. No significant difference was observed in all parameters for the bladder and rectum between the cFF-VMAT and FFF-VMAT groups.

**Table 2:** Dosimetric comparison for OARs between cFF-VMAT and FFF-VMAT VMAT, Volumetric-modulated arc therapy; cFF, conventional flattening filter; FFF, flattening filter-free; OAR, organs at risk; DVH, dose-volume histogram; SD, standard deviation.

DVH parameters	cFF-VMAT (mean ± SD)	FFF-VMAT (mean ± SD)	p-value
Bladder			
V10 (%)	68.4 ± 29.7	68.5 ± 29.8	0.838
V20 (%)	58.4 ± 31.2	58.5 ± 31.4	0.799
V30 (%)	46.1 ± 28.0	46.3 ± 28.1	0.508
V40 (%)	34.2 ± 21.7	34.4 ± 22.0	0.386
V50 (%)	25.0 ± 16.4	25.2 ± 16.7	0.285
V60 (%)	17.0 ± 11.3	17.0 ± 11.3	0.333
Dmean (Gy)	29.7 ± 14.7	29.7 ± 14.8	1.000
Rectum			
V10 (%)	69.7 ± 7.6	69.9 ± 7.6	0.386
V20 (%)	54.8 ± 6.1	55.7 ± 7.2	0.508
V30 (%)	31.3 ± 6.4	31.3 ± 6.5	0.959
V40 (%)	20.1 ± 4.8	20.1 ± 5.0	0.879
V50 (%)	13.1 ± 3.6	13.0 ± 3.7	0.241
V60 (%)	6.6 ± 2.3	6.3 ± 2.4	0.075
Dmean (Gy)	23.9 ± 2.9	23.9 ± 3.1	0.476

Technical parameters

**Figure 3:**
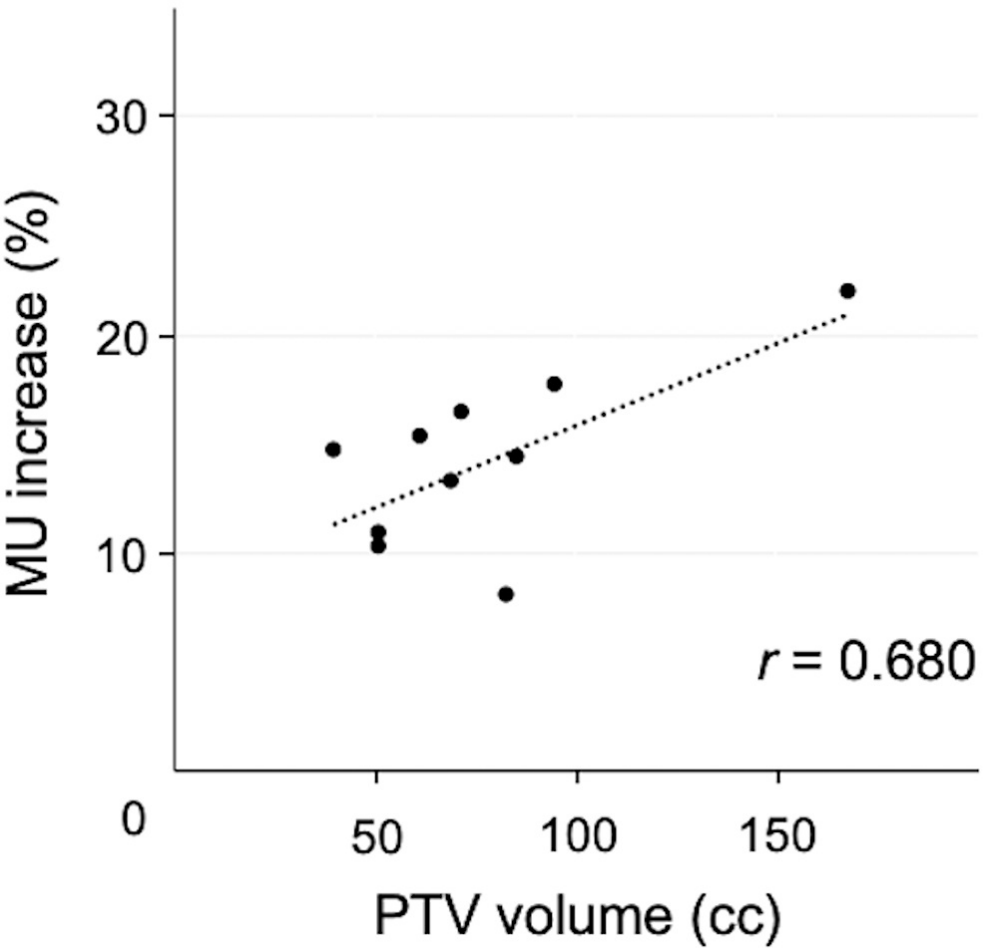
Scatter plots of planning target volume (PTV) volume and monitor units (MU) in volumetric-modulated arc therapy with flattening filter-free beams (FFF-VMAT) The correlation between PTV volume and technical parameters was evaluated by the Pearson correlation coefficient. The PTV volume and percentage of MU increases are moderately correlated (r = 0.680).

The technical parameters are summarized in Table 3. The mean PTV volume was 78.0 ± 36.2 cc. The mean values of MU were 686 ± 52 and 784 ± 80 in cFF-VMAT and FFF-VMAT, respectively. MU was significantly higher in FFF-VMAT than that in cFF-VMAT (p < 0.001). MU and PTV volumes were strongly correlated in both the cFF-VMAT and FFF-VMAT groups (r = 0.900 and 0.930, respectively). The mean percentage of MU increase was 14.2 ± 4.0% in the FFF-VMAT plans compared to cFF-VMAT. The percentage of MU increase was moderately correlated with the PTV volume (r = 0.680) as shown in Figure 3.

**Table 3:** Pearson's correlation coefficient with PTV volume PTV, Planning target volume; MU, monitor unit; BOT, beam-on time; VMAT, volumetric-modulated arc therapy; cFF, conventional flattening filter; FFF, flattening filter-free; SD, standard deviation.

Parameters	Mean ± SD	Pearson's r with PTV volume
MU		
cFF-VMAT	686 ± 52	0.900
FFF-VMAT	784 ± 80	0.930
MU increase (%)	14.2 ± 4.0	0.680
BOT		
cFF-VMAT (sec)	97.0 ± 6.6	0.850
FFF-VMAT (sec)	72.9 ± 1.4	0.919
BOT decrease (%)	24.6 ± 3.8	0.740

The mean BOT was 97.0 ± 6.6 s and 72.9 ± 1.4 s for cFF-VMAT and FFF-VMAT, respectively. The BOT was significantly shorter in FFF-VMAT than that in cFF-VMAT (p < 0.001).

**Figure 4:**
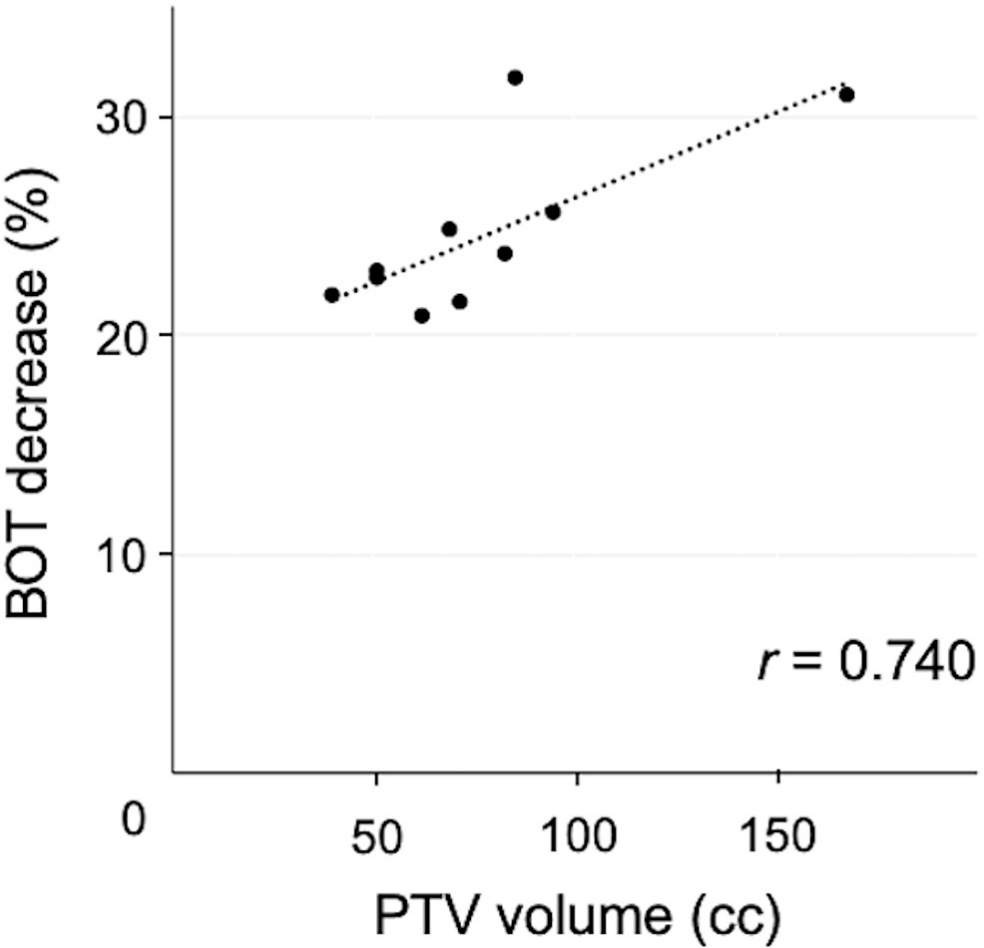
Scatter plots of planning target volume (PTV) and beam-on time (BOT) in volumetric-modulated arc therapy with flattening filter-free beams (FFF-VMAT) The correlation between PTV volume and technical parameters was evaluated by the Pearson correlation coefficient. The PTV volume and percentage of BOT decrease were strongly correlated (r = 0.740).

The BOT and PTV volumes were strongly correlated in both the cFF-VMAT and FFF-VMAT groups (r = 0.850 and 0.919, respectively). The mean percentage of BOT decrease was 24.6 ± 3.8% in the FFF-VMAT plans compared to cFF-VMAT. The percentage of BOT decrease was strongly correlated with PTV volume (r = 0.740) as shown in Figure 4.

## Discussion

In this study, we compared cFF-VMAT and FFF-VMAT treatment plans for prostate cancer. All DVH parameters of PTV and OARs were not significantly different. In FFF-VMAT, MU was significantly higher, and the BOT was significantly shorter than those in cFF-VMAT. This is a rare report comparing cFF-VMAT and FFF-VMAT in moderate hypofractionated RT for prostate cancer.

Several studies have demonstrated that the difference of dose distribution in the target volume was not significant between FFF-VMAT and cFF-VMAT. Stereotactic RT using FFF-beam for lung cancer with a large dose fraction of 10-12.5 Gy showed that the dose distribution for target volume was comparable to the RT plan with conventional flattening filter beam [25-27]. PTV coverage was also comparable in a study of FFF-VMAT for nasopharyngeal cancer [28]. A study of multiple treatment sites revealed that D2 and HI of PTV were significantly higher in FFF-VMAT than those in cFF-VMAT, but they were negligible [29]. Similar results have been reported for FFF-VMAT for prostate cancer in some studies. In studies on the use of ultrahypofractionated FFF-VMAT for prostate cancer, the CI and HI of the target volume were shown to be similar to that of the cFF-VMAT plan [18,19]. In addition, the target volume coverage was also comparable between cFF-VMAT and FFF-VMAT in the conventional fraction for prostate cancer [20]. In the present study using moderate hypofractionated irradiation, an equivalent dose distribution for PTV was obtained from each treatment group. Therefore, it was suggested that the dose distribution in the target volume was generally equivalent in FFF-VMAT, regardless of the treatment site or the dose fractionation.

Some studies demonstrated that DVH parameters of OARs were not also different between FFF-VMAT and cFF-VMAT. In a study of stereotactic RT using FFF-VMAT for lung cancer, doses in the lung, spinal cord, and heart were not different compared with cFF-VMAT [27]. A study of FFF-VMAT for prostate, head and neck, and brain cancers demonstrated similar doses of OARs with cFF-VMAT [29]. In addition, comparable dose distribution on OARs was observed with FFF-VMAT for prostate cancer [18-20], which was consistent with that of our study. Conversely, in FFF-VMAT of nasopharyngeal carcinoma, doses for the brainstem, spinal cord, and salivary glands were significantly higher than those in cFF-VMAT [28]. Therefore, several studies have demonstrated that FFF-VMAT generally provides a similar dose distribution for OARs to cFF-VMAT; however, there may be differences depending on the treatment site, and further study is warranted.

Several studies have shown that MU is higher in FFF-VMAT than that in cFF-VMAT. Additional MU is necessary to gain a uniform dose distribution with an inhomogeneous beam profile without FF [20]. A study on stereotactic RT for lung cancer showed that MU was increased by 5% with FFF-VMAT as compared to cFF-VMAT [27]. In a study of nasopharyngeal carcinoma, MU was increased by 7% with FFF-VMAT as compared to cFF-VMAT [28]. MU was elevated by 10%-25% in FFF-VMAT as compared to cFF-VMAT in prostate cancer [18-21]. The present study also showed similar results, i.e., MU values were significantly higher in FFF-VMAT and increased by 14.2% than cFF-VMAT. Hence, it was suggested that MU was higher in FFF-VMAT than that in cFF-VMAT, regardless of the treatment site or the dose fractionation.

Arslan et al. compared cFF-IMRT and FFF-IMRT according to the PTV volume [30]. MU was significantly higher in the larger PTV volume group than that in the smaller PTV volume. In that study, comparable dose distribution and higher MU were also demonstrated in FFF-IMRT. Vieillevigne et al. reported on the effect of PTV volume on cFF-VMAT and FFF-VMAT to a virtual phantom [31]. They showed that an increase in PTV volume was significantly correlated with the percentage of MU increase in FFF-VMAT. The present study also demonstrated a moderate correlation between PTV volume and percentage of MU increase. Although MU was suggested to be higher in patients with larger PTV volume, reports on the relationship between PTV volume and MU in FFF-beam are still limited, and further studies are needed.

One of the advantages of FFF-VMAT is shortening the treatment time. In stereotactic RT for lung cancer, FFF-VMAT reduced the irradiation time by approximately 50%-80% than cFF-VMAT [26,27,32]. In the treatment of nasopharyngeal carcinoma using a total dose of 70 Gy in 31 fractions, the irradiation time was not reduced [28]. In that study, according to the small dose fraction delivered with two full arcs of beams, the irradiation time was speculated to be limited by the gantry rotation and movement speed of the multi-leaf collimator. In addition, the large target volume for nasopharyngeal cancer may have also affected the irradiation time. Several studies of FFF-VMAT for prostate cancer reported an 8%-25% reduction in irradiation time [18-21]. Similar results were obtained in the present study, where FFF-VMAT reduced the BOT by 24.6%. Intrafractional motion cannot be ignored in the RT for prostate cancer [20]. Therefore, it is expected that intrafractional motion can be suppressed by shortening the irradiation time in prostate cancer by the use of FFF-VMAT. In addition, shortening the treatment time is patient-friendly and is also expected to improve the treatment throughput.

There are several limitations of this study. First, this study was a small-number survey in a single facility. Second, we investigated only the Monaco treatment planning system. In addition, various VMAT plan parameters, such as statistical uncertainty, minimum segment width, and fluence smoothing, can be modified in the Monaco treatment planning system. Several studies suggested that MU and irradiation time can vary by adjusting these parameters [33-35]. Several anatomical factors such as the size of the target volume and location of OARs may affect dose distribution and MU. Further studies are needed to clarify the similarities and differences between FFF-VMAT and cFF-VMAT.

## Conclusions

In this study, we compared the RT plans between cFF-VMAT and FFF-VMAT for prostate cancer. The dose distribution of FFF-VMAT was comparable to that of cFF-VMAT. The higher MU and shorter BOT were shown in FFF-VMAT compared to cFF-VMAT.
